# Surgery

**Published:** 1859-03

**Authors:** 


### Bi-Monthly Periscope.

### SURGERY.

1.—EPILEPSY FOR THIRTY-TWO YEARS IN A MAN AGED FORTY-FOUR, WITH DISCOLORATION OF THE SKIN FROM NITRATE OF SILVER; OPERATION OF CASTRATION. Under the care of *Mr. Holthouse.*—Among the causes of epilepsy mentioned by various writers, extreme sexual excesses are considered as not the least important. They would appear to have much influence on the frequency of the fits, as is shown in the narrative of the following case, the notes of which were taken by Mr. H. Ponsonby Adair, house-surgeon to the hospital. There are cases on record in which castration has been resorted to as a means of relief. In one reported by Mr. J. P. Frank, the *aura epileptica* began in the testicle, and it is asserted that a permanent cure followed castration.

This operation is much practiced at the present day among the Eastern nations, for the sole purpose of depriving their slaves of manhood; and Mr. Curling informs us, in his work on the "Diseases of the Testis," that in Italy it was once frequently performed, on account of its effects on the vocal organs.

Eli B——, aged forty-four, widower, native of the United States, bookseller, was admitted into Luke ward, in the Westminster Hospital, on the 4th of January, under the care of Mr. Holthouse, in order to have the operation of castration performed for the cure of epilepsy.

The patient is one of fourteen children, of whom eleven are living and healthy; his father is alive, aged eighty-four, and his mother died at eighty. There is no insanity in his family, nor is any member of it afflicted with epilepsy. He was a healthy child till he was ten years of age, when he commenced to practice masturbation, and soon after had an epileptic fit, in which he bit his tongue. This was followed by severe pain in the head, and incapacity for exertion next day. The fits recurred every three or four weeks. They came on suddenly, without any premonitory symptoms. During the first two years he took "skull-cap tea," without effect; his diet was also regulated. He still continued to practice self-abuse, and did not finally relinquish it till he was twenty-two, about the time when he began to take nitrate of silver. For two years he tried homœopathy, the fits increasing in severity. He was at school up to the age of fifteen, when he tried a sea-voyage, but without benefit. Having returned, he sailed for South America, where he remained for two years, the fits being as frequent as before. While at New York he contracted gonorrhœa, having been accustomed to frequent sexual intercourse from the age of sixteen, in addition to the habit of self-abuse. He remained in New York for a few months, trying various remedies, among them sulphate of zinc, but without relief. He went again to the South for a few months, and upon his return he placed himself under the care of Dr. Kissam, (his brother-in-law,) who prescribed nitrate of silver, in doses of one-eighth of a grain, three times daily, and in two months it was increased to half a grain. Very soon after he began to take this remedy, the severity and frequency of the fits began to decrease, and he was so convinced of its efficacy, that he continued its use for about eight months, against the advice of Dr. Kissam, who feared it might affect his skin, which, indeed, it did to some extent, giving it a blue tint. At the end of this time, the fits left him for a period of two years, having gradually decreased in frequency under the use of the nitrate of silver. From the time of his contracting gonorrhœa till his marriage, he abstained altogether from sexual intercourse and the habit of self-abuse, so that during the whole time that he was taking the nitrate of silver he had no extraneous sexual excitement; yet during this period he says that he was constantly troubled with nocturnal erections, and frequent seminal emissions. Being now twenty-four years of age, he married, shortly after which he again became addicted to sexual excesses. He left his wife and his business for several months, and traveled; the fits, however, recurred every three or four weeks, and were very severe. On his return his wife died, and he remained a widower six years, abstaining altogether from sexual excesses, although frequently troubled with erections. During the six years he broke his arm, several fingers, and his leg twice, while in the fits. At the age of thirty he married a second time, the fits having increased in number and severity. He was often compelled to send his wife into the country for a day or two, in order to avoid sexual excitement. The fits now recurred daily. His wife died a year after marriage. After this he again abstained from sexual excesses. Dr. Horace Green, of New York, now cauterized his larynx daily with nitrate of silver, and at the end of three or four months he would be free from fits for nineteen days; when they did recur, they were so slight that he scarcely lost consciousness, and did not fall down. This plan of treatment was pursued for two or three years, at the end of which time he became attached to another young woman, which revived all his old amatory feelings, and the fits began to increase in frequency, recurring at intervals of fourteen days, when they would continue daily for a week, and then cease for fourteen days more. Galvanism was now tried, with some slight beneficial effect. Next arsenic, in the form of Fowler's solution, which he continued till the fits recurred daily, and he became so prostrate that he was confined to his bed. For a long time he took iron to neutralize the effects of the arsenic, but for months he was compelled to walk on crutches. He came to England two years ago, to have tracheotomy performed by Dr. Marshall Hall, who had advised it when he saw the man in America. Dr. Hall died soon after the man's arrival, and he went to Paris, and was under the care of M. Nélaton. Afterwards, he placed himself under the care of M. Trousseau, who gave him belladonna, which affected his vision, but not his fits. Dr. de Lasiauve next treated him with camphor for four months, without effect. He returned to England, and was under Mr. Simon, at St. Thomas's Hospital, in order to have castration performed, in which he had great faith, for he attributed his fits chiefly to sexual excitement, which still troubled him much; but his wish was not acceded to. He took bromide of potassium without any benefit, and then the nitrate of silver for two or three months, in half-grain doses three times a day. The skin became darker than before, and the fits recurred daily. He next went to Germany, and was there sounded for a stone in the bladder on account of frequent micturition, which he has had since infancy. No calculus was present. He was an inmate of the hospitals of Vienna, Prague, and Dresden. He left the latter in October, 1858, and was admitted into the Westminster Hospital, under Dr. Radcliffe, on the 30th of the month, and remained in two months, during which time he took quinine and iron, and camphor, but without avail. Since his second wife's death he has entirely abstained from sexual intercourse, though he has been constantly troubled with nocturnal erections, and occasional seminal emissions, and these continued up to the time when he came under the care of Mr. Holthouse, to whom he applied to perform castration, which, after much deliberation, he consented to do; and it was performed upon both testicles on the 4th of January, 1859, under the influence of chloroform. Two or three hours afterwards, there was considerable hemorrhage, which was checked by the application of cold. He had one fit during the hemorrhage. His face has a bluish-slate tinge, which pervades the body, but the color is darkest on the face. His fits are of the rotary kind, preceded by a sudden scream, and lasting not more than a minute, and when over he is quite himself again. In the fit which he had in bed after the operation, he did not scream, but merely struggled violently.

January 5th.—He had another fit this morning.

6th.—The fit recurred early this morning.

7th.—At four this morning another fit occurred. He says that after his second marriage the fits frequently followed immediately on the act of connection.

8th.—Has had no fit at all to-day.

9th.—Had a very slight attack this morning, scarcely more than a giddiness for a minute. Altogether, since the operation, the fits have been exceedingly mild.—*Lancet*, January 22d, 1859.

2. OBSERVATIONS ON THE TREATMENT OF SOME OF THE SYMPTOMS OF SYPHILIS. By *M. Hervieux.* (*Bulleten de Thérapeutique*, tome liv., pp. 441 and 529.)

1. *Phagedœnic Chancre.*—M. Hervieux observes that it is very natural that a disease which produces such rapid local destruction should have been met by means rivaling it in energy and celerity of action, such as the butter of antimony, the various forms of caustic, the actual cautery, etc. But, although all those means have been successful in some cases, it is certain that they have still oftener failed, or they would not have been so generally abandoned. There is one means, however, which, in the hands of M. Ricord, has proved of indubitable advantage, viz., the *carbo-sulphuric paste*, prepared by mixing sulphuric acid with powdered vegetable charcoal in sufficient proportions to form a semi-solid paste. When applied to the chancre this soon dries, forming a black crust, which intimately adheres to the tissues, and only falls off after several days, leaving a clean sore, or even, in some cases, a cicatrized surface. In the author's practice, pure *tincture of iodine*, applied at the commencement, has proved to be the best means of arresting the progress of the disease. It induces generally a burning pain, the intensity and duration of which are in proportion to the extent and depth of the chancre, as also to the sensibility of the individual and of the parts affected. Very well borne by some patients, the pain induces in others the most horrible torment. Chloroform would in such nervous and irritable subjects save this suffering. The pain, upon an average, lasts half an hour. In simple, uncomplicated cases, two applications, made by means of a pencil, after an interval of twenty-four hours, generally arrest the progress of the blood. If, however, the chancre be complicated with gangrene, hospital gangrene, or diphtheria, four, five, or even six applications may be required. But when two or three of these seem to be without any effect, there is no use going on with the iodine, and a solution of nitrate of silver (five parts to thirty) should be substituted. When the iodine treatment has been followed, M. Hervieux has never known the worst form of phagedæna persist beyond a week.

2. *Suppurating Bubo.*—The author has never himself treated bubo by small, single, or multiple openings, but he has met with cases which have been so treated, and which, two or three months afterwards, have exhibited fistulous tracks, extensive detachment, thinning and changes in the skin, together with an utter indisposition to heal. After waiting two or three weeks in vain for the spontaneous closure of these fistulæ, he has had to lay them freely open. The prevention of deformity by these small apertures, as proposed by Vidal, is frequently not attained, for not only may fistulous tracks become established, but the apertures themselves may become transformed into chancrous ulcerations. As a general rule, M. Hervieux makes a large opening, and that as early as possible, cicatrization taking place most rapidly under these circumstances. When the opened bubo is transformed into a strumous or chancrous ulcer, or the two combined, with the possible complication of phagedænism, he treats it by the application of the tincture of iodine or solution of the nitrate of silver, washing it out also with chlorine lotions several times a day; and he has never found any ulcer resisting treatment longer than six weeks, the majority becoming healed in from eight to fifteen days.

3. *Condylomata* (*Plaques muqueuses.*)—Although the author believes the practice he recommends under the former heads may require additional confirmation from more extensive practice than his own, in the matter of condylomata he can speak more positively. If the solution of nitrate of silver is not an actual specific, it acts with such rapidity, certainty, and efficacy, as to call for the highest recommendation. However confluent they may be and whatever extent of surface they may occupy, however infectious the discharge they give out, and even when they have attained a certain amount of thickness, provided that they are not too hypertrophied and have not undergone some of the transformations they are susceptible of, they will wither, die away, and disappear in the course of some days, if every part be painted daily with a pencil dipped in a solution of the nitrate, five parts to thirty of water. Baths should be simultaneously used, seeing the part which dirt habitually takes in the production of this accident. Repeated trials have convinced the author that this success is quite independent of internal treatment. When, however, the condylomata have become transformed into a vast vegetating surface, of great thickness, the nitrate ceases to be of avail; and in one aggravated case mentioned, the pure nitric acid, repeatedly applied, was of service.

4. *Syphilides.*—Under this head the author gives the results of his trial, in ten cases, of M. Cullérier's plan of treating syphilitic eruptions by blisters applied to the chest. Although at first prepossessed against it, he now speaks highly in its favor. Excluding the slight roseolar forms, which get well of themselves, the author oftenest employed blistering in the papular form of the disease, and that is the form in which the remedy best succeeded. A single blister will exert a notable modification on chronic papular syphilides, which have existed during several months. One case of syphilitic lichen, which had lasted a year, and for which all kinds of active internal treatment had been tried, disappeared in the course of a week, during which three large blisters were successively applied to the anterior and posterior surfaces of the thorax. The squamous form resisted their action more, but still in two cases of psoriasis undoubted amendment was observable, and in a fortnight the scales were detached. In the pustular form, some cases of syphilitic acne were rapidly cured. M. Hervieux has not tried blistering in syphilitic impetigo of the face and hairy scalp, having found the application of the nitrate of silver solution, after poulticing off the crusts, very efficacious, even in very inveterate cases.—*British and Foreign Medico-Chirurgical Review*, January, 1859.

3. ON THE TREATMENT OF HERNIA BY ELECTRICITY. By *Dr. Clemens.* (*Deutsche Klinik*, No. 34.)—This paper is the first of a series the author intends publishing upon the therapeutical application of electricity—a subject that has engaged his attention for some years past. He first employed this agent in the treatment of inguinal hernia, in 1850, and has frequently had recourse to it since then. The hernia being reduced, and the patient placed in the semi-recumbent position, the ball of the conductor is carried as far into the hernial canal as possible, and the application of the electricity continued during five minutes, its power being increased day by day. After a few *séances* the mouth of the ring becomes diminished in size, the finger is introduced with more difficulty, and the hernia will not descend so easily as heretofore. The electricity, too, exerts a very beneficial effect upon the peristaltic intestinal motions, augmenting and regulating these, and thus preventing the same relaxed portion of intestine from always lying opposite the hernial aperture. A state of obstinate constipation becomes changed for one of regular action, and many old disordered conditions of the abdominal cavity become relieved. When the hernia has been recently produced, no means act with so much certainty and rapidity; and a case is referred to of a young man who acquired double inguinal hernia during an effort to raise a heavy burden, and which was completely cured after twenty *séances*, although these were not commenced until a week after the accident. Under its agency recent hernia is rapidly returned: but the author has not yet tried it in a case of complete incarceration. Among the twenty-seven cases in which it has been resorted to, none have manifested the slightest ill consequences. Dr. Clemens prefers static electricity to galvanism, and administers it by means of the Leyden phial.

Another application of electricity by the author consists in a galvanic hernia truss, for a description of the construction of which we must refer to his paper. By its agency a feeble but constant galvanic stream is kept applied to the ring, and large hernias soon become easily retained which before had resisted the largest trusses and the strongest springs. Of late, the author has constructed a pile of silver and copper coins, and the effects of so small an apparatus have often surprised him.—*British and Foreign Medico-Chirurgical Review*, January, 1859.

